# Monoamine oxidase-A (MAO-A) low-expression variants and increased risk of *Plasmodium vivax* malaria relapses

**DOI:** 10.1093/jac/dkae196

**Published:** 2024-06-13

**Authors:** Maria Carolina Silva De Barros Puça, Danielle Fonseca Rodrigues, Yanka Evellyn Alves Rodrigues Salazar, Jaime Louzada, Cor Jesus Fernandes Fontes, André Daher, Dhélio Batista Pereira, José Luiz Fernandes Vieira, Luzia Helena Carvalho, Cristiana Ferreira Alves de Brito, José Pedro Gil, Tais Nobrega de Sousa

**Affiliations:** Molecular Biology and Malaria Immunology Research Group, Instituto René Rachou, Fundação Oswaldo Cruz (FIOCRUZ), Belo Horizonte, Minas Gerais, Brazil; Molecular Biology and Malaria Immunology Research Group, Instituto René Rachou, Fundação Oswaldo Cruz (FIOCRUZ), Belo Horizonte, Minas Gerais, Brazil; Molecular Biology and Malaria Immunology Research Group, Instituto René Rachou, Fundação Oswaldo Cruz (FIOCRUZ), Belo Horizonte, Minas Gerais, Brazil; Universidade Federal de Roraima, Boa Vista, Roraima, Brazil; Universidade Federal do Mato Grosso, Faculdade de Medicina, Departamento de Medicina Interna, Cuiabá, Mato Grosso, Brazil; Vice Presidency of Research and Biological Collections, Fundação Oswaldo Cruz (FIOCRUZ), Rio de Janeiro, Rio de Janeiro, Brazil; Centro de Pesquisa em Medicina Tropical de Rondônia, CEPEM, Porto Velho, Rondônia, Brazil; Universidade Federal do Pará, Faculdade de Farmácia, Laboratório de Toxicologia, Belém, Pará, Brazil; Molecular Biology and Malaria Immunology Research Group, Instituto René Rachou, Fundação Oswaldo Cruz (FIOCRUZ), Belo Horizonte, Minas Gerais, Brazil; Molecular Biology and Malaria Immunology Research Group, Instituto René Rachou, Fundação Oswaldo Cruz (FIOCRUZ), Belo Horizonte, Minas Gerais, Brazil; Department of Microbiology, Tumor and Cell biology, Karolinska Institutet, Solna, Sweden; Molecular Biology and Malaria Immunology Research Group, Instituto René Rachou, Fundação Oswaldo Cruz (FIOCRUZ), Belo Horizonte, Minas Gerais, Brazil; Department of Microbiology, Tumor and Cell biology, Karolinska Institutet, Solna, Sweden

## Abstract

**Objectives:**

Primaquine is essential for the radical cure of *Plasmodium vivax* malaria and must be metabolized into its bioactive metabolites. Accordingly, polymorphisms in primaquine-metabolizing enzymes can impact the treatment efficacy. This pioneering study explores the influence of monoamine oxidase-A (MAO-A) on primaquine metabolism and its impact on malaria relapses.

**Methods:**

Samples from 205 patients with *P. vivax* malaria were retrospectively analysed by genotyping polymorphisms in *MAO-A* and cytochrome P450 2D6 (*CYP2D6*) genes. We measured the primaquine and carboxyprimaquine blood levels in 100 subjects for whom blood samples were available on the third day of treatment. We also examined the relationship between the enzyme variants and *P. vivax* malaria relapses in a group of subjects with well-documented relapses.

**Results:**

The median carboxyprimaquine level was significantly reduced in individuals carrying low-expression *MAO-A* alleles plus impaired CYP2D6. In addition, this group experienced significantly more *P. vivax* relapses. The low-expression *MAO-A* status was not associated with malaria relapses when CYP2D6 had normal activity. This suggests that the putative carboxyprimaquine contribution is irrelevant when the CYP2D6 pathway is fully active.

**Conclusions:**

We found evidence that the low-expression MAO-A variants can potentiate the negative impact of impaired CYP2D6 activity, resulting in lower levels of carboxyprimaquine metabolite and multiple relapses. The findings support the hypothesis that carboxyprimaquine may be further metabolized through CYP-mediated pathways generating bioactive metabolites that act against the parasite.

## Background

*Plasmodium vivax* is a widespread malaria species, contributing significantly to the overall malaria burden.^1^ The 8-aminoquinoline primaquine is a key drug used for the radical cure of *P. vivax* malaria, targeting latent hypnozoites in the liver.^2^ It is also recommended to interrupt *Plasmodium falciparum* transmission due to its activity against mature gametocytes.^3^ A major concern about the use of the 8-aminoquinolines such as primaquine or its synthetic analogue tafenoquine is related to the risk of oxidative haemolysis in individuals with glucose-6-phosphate dehydrogenase (G6PD) deficiency, which is estimated to affect around 8% of the population across *P. vivax* endemic countries.^2^

Primaquine is metabolized by two key enzymes in the liver, cytochrome P450 2D6 (CYP2D6) and monoamine oxidase-A (MAO-A).^4^ Both the therapeutic efficacy and toxicity of primaquine (PQ) have been attributed to its hydroxylated metabolites (OH-PQm) produced mainly by CYP2D6.^3–5^ The oxidation and redox cycling of OH-PQm into its corresponding quinoneimine forms can produce hydrogen peroxide (H_2_O_2_) and lead to parasite killing.^6,7^ MAO-A is implicated in the formation of the most predominant metabolite of primaquine, carboxyprimaquine.^3,4,8^ The oxidative deamination of primaquine through MAO-A pathway is responsible for its short elimination half-life (∼4–6 h).^9^ Albeit carboxyprimaquine does not show direct activity against the parasite, there is evidence that carboxyprimaquine may be metabolized through phase I CYP-mediated reactions with concomitant formation of hydroxylated and quinoneimine metabolites.^9^

The *MAO-A* proximal promotor sequence is polymorphic, containing a variable number of tandem repeat polymorphisms (uVNTR). It consists of a 30 bp sequence, present in 2–5 copies, which has been shown to influence transcription.^10^*MAO*-*A* is an X-linked gene, meaning that males are considered hemizygotes. While the role of X chromosome inactivation in regulating MAO-A levels remains uncertain, there is evidence of higher MAO-A activity in heterozygous individuals.^11^ The gene *CYP2D6* is highly diverse, with over 150 defined alleles and a wide range of phenotypes, from complete dysfunction to ultrarapid metabolism. Previous studies have shown that low-activity CYP2D6 variants reduce the hypnozoitocidal efficacy of primaquine, resulting in repeated relapses in *P. vivax* malaria.^3,12,13^ Here, we interrogated to what extent MAO-A variants could contribute to the primaquine efficacy.

## Methods

Samples from 205 subjects infected by *P. vivax* were retrospectively analysed. The first part included 100 subjects from Boa Vista, Roraima State, who were mostly involved in gold mining activities and had a high risk of infection by *Plasmodium* spp. Their blood levels of primaquine and carboxyprimaquine were measured on the third day of treatment. We also evaluated the association between the enzymatic variants and relapses in 105 subjects residing in areas with unstable transmission or no active malaria transmission: Souza in Minas Gerais State (*n* = 16), Cuiabá in Mato Grosso State (*n* = 40) and Porto Velho in Rondônia State (*n* = 49). Most samples were from cross-sectional studies, except for Porto Velho, which also included samples from a drug efficacy study. The study areas and participants have been described previously^14–16^ and are described in the Supplementary Data. The ethical and methodological aspects of the study were approved by the Ethics Committee of Research Involving Human Subjects of Institute René Rachou/Fiocruz (report no. 2.803.756). All participants signed a written informed consent form, and the next of kin, caretakers or guardians signed on behalf of minors/children enrolled in the study.

Most participants were adults with a median age of 34 years (IQR, 25–46); none reported antimalarial use in the preceding 30 days or a history of chronic conditions (such as severe cardiac, hepatic or renal disorders). All patients were treated with a combination of a schizonticidal drug such as chloroquine (total dose of 25 mg/kg over 3 days) and primaquine (total dose of 3.5 mg/kg over 7 days).

The number of *P. vivax* malaria episodes for each participant was obtained from the Epidemiological Surveillance System for Malaria (SIVEP-Malaria). *P. vivax* recurrence was defined as a new episode diagnosed microscopically, occurring within an interval ranging from 29 to 180 days after the initial episode. For participants of the drug efficacy trial, parasite relapses were assayed within 63 days of follow-up.

The uVNTR polymorphism in MAO-A was genotyped by conventional PCR followed by fragment analysis with 1 bp precision through capillary electrophoresis.^10^ Heterozygous women carrying both high- and low-expression MAO-A alleles were classified as extensive metabolizers. *CYP2D6* polymorphisms (C1584G, C100T, C1023T, G1846A, C2580T, G2988A, G3183A, G4180C and 2615_2617delAAG) and the copy number of the gene were assayed by qPCR.^13^ These are common genetic variants in Brazilians associated with reduced drug metabolism.

Primaquine and carboxyprimaquine were quantified on the third day of treatment using a reversed-phase HPLC system with a diode array detector (Flexar System—Perkin Elmer Inc., Boston, MA, USA). The measurement involved liquid–liquid extraction from blood spots on filter paper, following the protocol previously described.^17^

Statistical analysis was performed using GraphPad Prism version 8.0.2 (GraphPad Software, San Diego, CA, USA). All tests were two-sided, and a *P* value of <0.05 was considered statistically significant.

## Results and discussion

**Figure 1.**
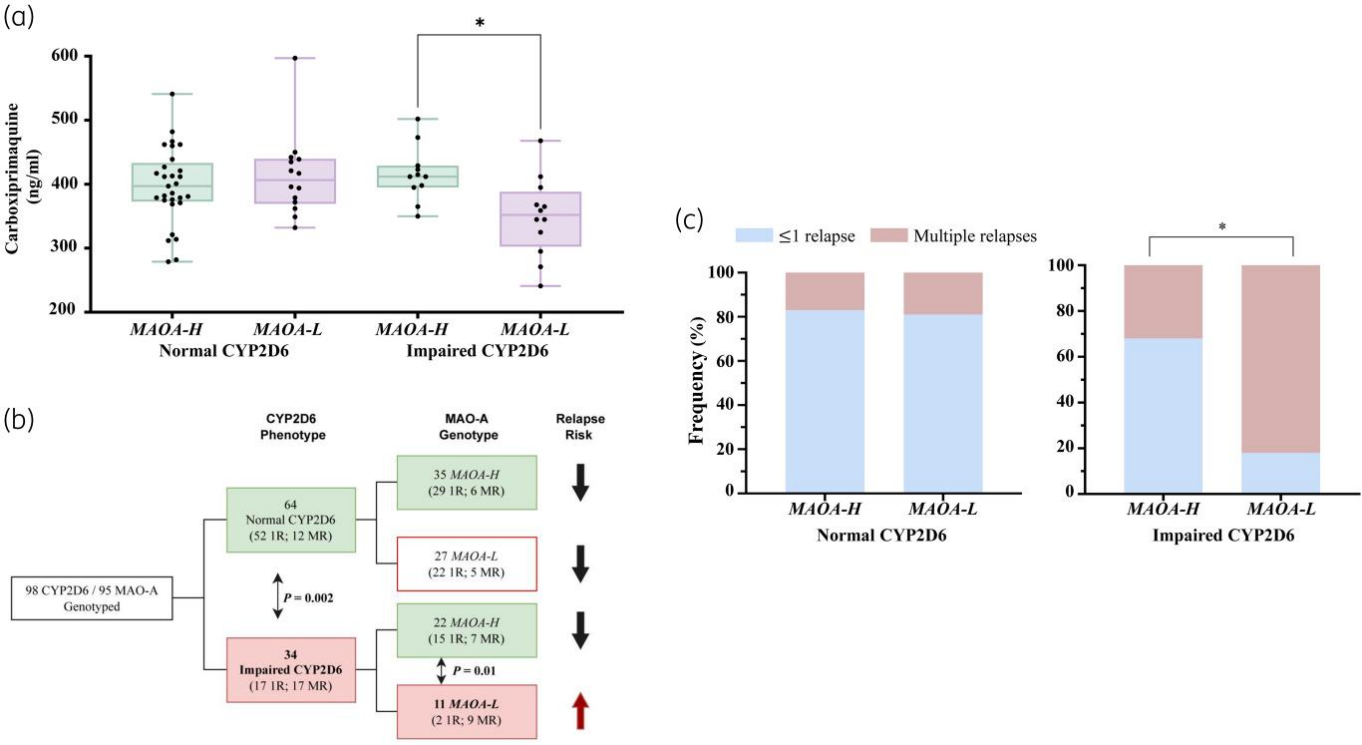
Drug blood levels and frequency of *P. vivax* malaria relapses according to MAO-A and CYP2D6 status. (a) Association between carboxyprimaquine blood levels and genetic status of CYP2D6 and MAO-A. Carboxyprimaquine was measured on Day 3 after the initiation of treatment in blood samples collected from participants from Boa Vista, Roraima State. The enzyme activity of MAO-A and CYP2D6 was inferred as follows: normal CYP2D6, normal/ultrarapid metabolizers (AS > 1·0); impaired CYP2D6, poor/intermediate metabolizers (AS ≤ 1·0); *MAOA-H*, MAO-A high expression; *MAOA-L*, MAO-A low expression. (b) Flowchart representing the number of individuals successfully genotyped in this study. For relapse analysis, we analysed individuals living in areas of unstable or without active transmission and subjects followed up for 2 months as part of a drug efficacy trial. (c) Frequency of malaria relapses among groups of individuals according to CYP2D6 and MAO-A activity status. ≤1R, non- or single relapse; MR, multiple relapses. This figure appears in colour in the online version of *JAC* and in black and white in the print version of *JAC*.

Initially, we measured the primaquine and carboxyprimaquine blood levels in 100 subjects for whom blood samples were available on the third day of treatment. Among them, 30% had impaired CYP2D6 activity inferred from genotype data (AS ≤ 1.0), and 31% carried low-expression MAO-A variants. No differences in primaquine levels were documented between high- and low-expression MAO-A variants [median = 179.0 ng/mL (IQR = 146.0, 232.0) and 188.0 ng/mL (119.0, 217.5), respectively, *P* = 0.907] in the context of impaired CYP2D6 activity (Figure S1, available as Supplementary data at *JAC* Online). Interestingly, the median carboxyprimaquine level was significantly reduced in the specific group of subjects carrying low-expression/activity alleles in both MAO-A and CYP2D6 (*P* = 0.034) (Figure 1a). These results point to a potential gene–gene interaction where the status of the *MAO-A* promoter region is critical, prompting the working hypothesis of a nuclear receptor-mediated mechanism.^18^

We additionally examined the relationship between the enzyme variants and *P. vivax* malaria relapses in a group of 105 individuals with well-documented relapses. Among them, 35% had impaired CYP2D6 activity (AS ≤ 1), and 38% of subjects carried the low-expression MAO-A variants (Table S1). In terms of therapy efficacy, impaired CYP2D6 activity was associated with multiple relapse episodes of *P. vivax*, when compared with the group of extensive/ultra-rapid CYP2D6 metabolizers [50% (17 out of 34) versus 19% (12 out of 64), *P* = 0.002] (Figure 1b), confirming the well-established importance of CYP2D6 activity and primaquine performance.

We next investigated whether the lower levels of carboxyprimaquine observed among patients carrying low-expression *MAO-A* alleles plus impaired CYP2D6 would impact primaquine efficacy. This group, in fact, experienced significantly more *P. vivax* relapses (*P* = 0.010) (Figure 1c), a relatively surprising observation, as carboxyprimaquine is generally considered pharmacologically inactive in malaria therapy. The low-expression *MAO-A* status was not associated with malaria relapses when CYP2D6 had normal activity (*P* > 0.999), suggesting that the putative carboxyprimaquine contribution is essentially irrelevant when the CYP2D6 pathway is fully active.

**Figure 2.**
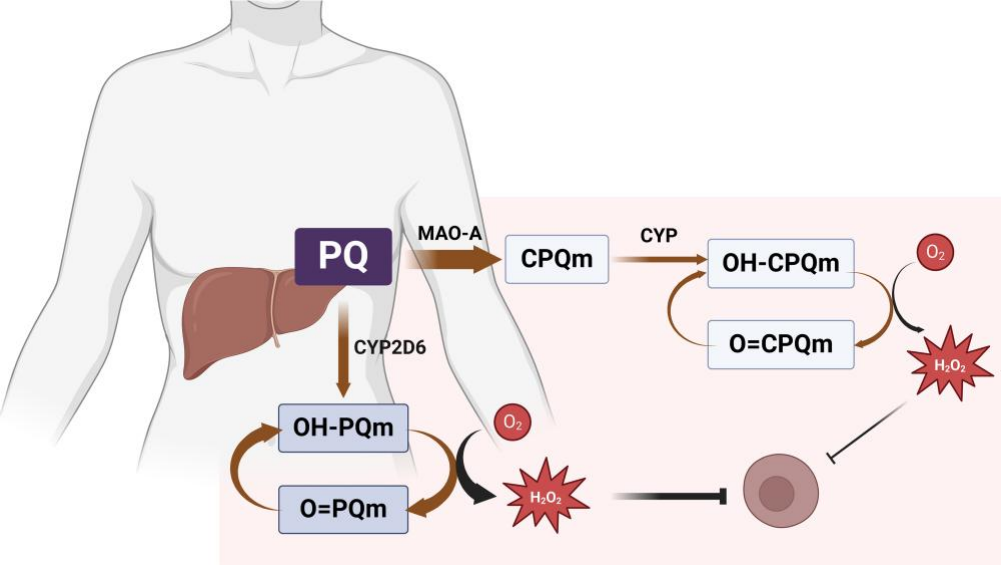
Schematic representation of the proposed mechanism of action of primaquine and carboxyprimaquine against liver hypnozoites. Primaquine (PQ) is metabolized into hydroxylated PQ metabolites (OH-PQm) by CYP2D6. OH-PQm undergoes spontaneous oxidation to quinoneimines (O = PQm) with concomitant generation of hydrogen peroxide (H_2_O_2_). Primaquine is converted to carboxyprimaquine, the most abundant plasma metabolite, mainly by MAO-A. Carboxyprimaquine produces its own set of quinoneimines that kill the parasite through redox cycling and oxidative damage. Created with BioRender.com. This figure appears in colour in the online version of *JAC* and in black and white in the print version of *JAC*.

For the moment, we propose as a hypothesis that carboxyprimaquine does have a role of protection by producing its own set of quinoneimines that kill the parasite through redox cycling and oxidative damage, ultimately impacting the drug’s anti-hypnozoite activity (Figure 2). The first evidence for hydroxylation of carboxyprimaquine followed by the formation of quinoneimines came from *in vitro* analysis using primary human hepatocytes.^19^ More recently, the presence of these compounds was confirmed by *in vivo* phenotyping of primaquine metabolites in plasma and urine from healthy human volunteers.^9^ Carboxyprimaquine metabolite production is smaller, but this is likely to be partially offset by a more extensive exposure, primarily considering carboxyprimaquine longer elimination half-life (∼15 h). Thus, less MAO-A activity would lead to a decreased rate of metabolite production and, hence, decreased protection. Nevertheless, this effect is likely to be secondary in the presence of fully active CYP2D6 activity, with primaquine metabolism through the MAO-A pathway alone not sufficient to produce enough active metabolites for effective protection against the parasite and prevent *P. vivax* relapses.

Our study has two main limitations. First, we could evaluate only a few subjects with altered activity of both enzymes (*n* = 11). A larger study is necessary to further elucidate the relationships between the spectrum of enzyme activities and relapses in *P. vivax* malaria. Second, we could not follow a standard approach related to the time of blood collection after drug intake, which may have contributed to the high variability observed in drug blood levels among subjects. Nonetheless, carboxyprimaquine level measurements should be less affected due to its longer elimination half-life.

### Conclusions

In conclusion, we present data supporting the potential importance of *MAO-A* promoter polymorphism as a factor to be considered, in addition to the well-established importance of *CYP2D6* pharmacogenetics in primaquine efficacy. Our findings can have a significant impact on malaria epidemiology, considering that approximately 35% of subjects may show low expression of MAO-A or impaired CYP2D6.^2,10^ Finally, it remains to be evaluated how individuals with genetic deficiency of G6PD can be affected by these enzyme variants concerning the risk of haemolytic toxicity of primaquine.

## Supplementary Material

dkae196_Supplementary_Data

## Supplementary data

Figure S1 and Table S1 are available as Supplementary data at *JAC* Online.
