# Supplementary material for: Monoamine oxidase-A (MAO-A) low-expression variants and increased risk of Plasmodium vivax malaria relapses
Source: J Antimicrob Chemother. 2024 Jun 13;79(8):1985–9. doi: 10.1093/jac/dkae196 (PMC11290872; doi:10.1093/jac/dkae196)
Supplement: dkae196_Supplementary_Data [file dkae196_supplementary_data.docx]

**SUPPLEMENTARY MATERIAL**

*Supplement to*: **Monoamine oxidase-A low-expression (MAO-A) variants and increased risk of Plasmodium vivax malaria relapses**

Maria Carolina Silva de Barros Puça, Danielle Fonseca Rodrigues, Yanka Evellyn Alves Rodrigues Salazar, Jaime Louzada_,_ Cor Jesus Fernandes Fontes, André Daher, Dhélio Batista Pereira, José Luiz Fernandes Vieira, Luzia Helena Carvalho, Cristiana Ferreira Alves de Brito, José Pedro Gil, Tais Nobrega de Sousa

**Supplementary Table 1**. Frequencies of predicted CYP2D6 phenotypes and MAO-A genotypes in the studied population from areas of unstable or without active transmission and subjects followed up for two months as part of a drug efficacy trial.

| **Enzyme variant** | **Enzyme activity/expression** | **Frequency, (n)** | **Non- or Single relapse, (n)** | **Multiple relapses, (n)** | ***P*-value** ^b^ |
| --- | --- | --- | --- | --- | --- |
| **CYP2D6** ^a^ |  |  |  |  |  |
| AS ≤ 1 | Impaired | 0.346 (34) | 0.173 (17) | 0.173 (17) | 0.002 |
| AS > 1 | Normal/High | 0.653 (64) | 0.531 (52) | 0.122 (12) |  |
| **MAO-A** |  |  |  |  |  |
| 2R/3R, 2R, 3R | Low | 0.376 (38) | 0.237 (24) | 0.138 (14) | 0.167 |
| 3R/4R, 3.5R/4R, 4R/4R, 3.5R, 4R | High | 0.623 (63) | 0.485 (49) | 0.138 (14) |  |

The two alleles carried by homo- or heterozygous women are separated by “/”.

^a^ Score activity (AS ≤ 1): gPM, poor metabolizers; gIM, intermediate metabolizers; gNM-S, normal-slow metabolizers; AS > 1: gNM-F, normal-fast metabolizers; gUM, ultrarapid metabolizers

^b^ Analysis of frequency distribution among groups: non- or single recurrence vs multiple recurrences (Fisher exact test). Number of subjects included in this analysis was 98 and 101, respectively, for CYP2D6 and MAO-A.

**
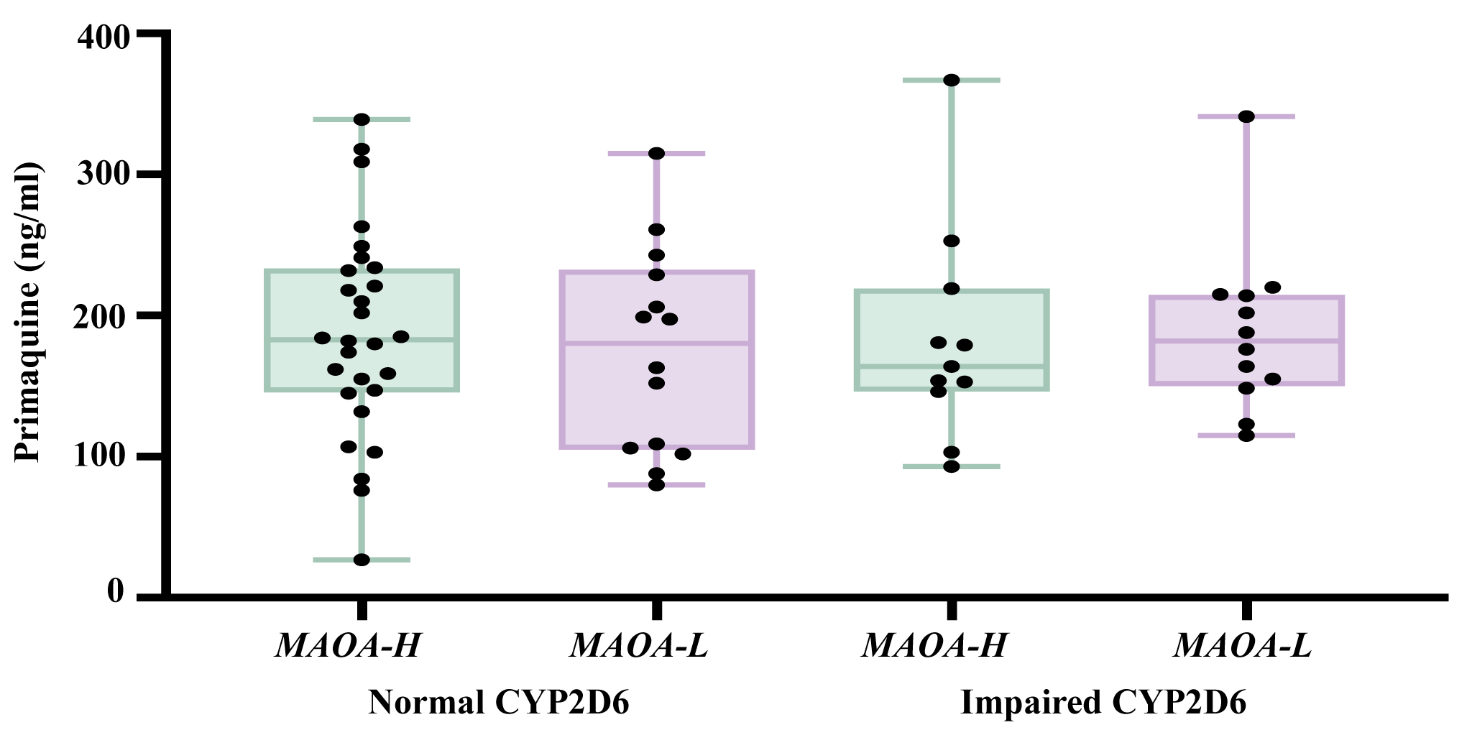
**

**Supplementary Figure 1. Primaquine blood levels according to MAO-A and CYP2D6 status.** Association between primaquine blood levels and genetic status of CYP2D6 and MAO-A. Primaquine was measured on day three after the initiation of treatment in blood samples collected from participants from Boa Vista, Roraima State. The enzyme activity of MAO-A and CYP2D6 was inferred as follows: Normal CYP2D6, normal/ultrarapid metabolizers (AS > 1·0); Impaired CYP2D6, poor/intermediate metabolizers (AS ≤ 1·0); *MAOA-H*, MAO-A high-expression; *MAOA-L*, MAO-A low-expression.

**METHODS**

# Study Site and Subjects

# This study included samples of *P. vivax-*infected patients from four areas in Brazil with different epidemiological characteristics, as described below. The details of the study areas and populations have been reported elsewhere.^1–4^

# *Areas of unstable or without active transmission and drug efficacy study*

# *Souza municipality, Minas Gerais State (MG)*: Samples (n = 16) were collected during an outbreak in 2003. Individuals infected with *P. vivax* were enrolled in the study if they resided in the outbreak area, were at least 15 years old, and, if women, the absence of pregnancy. Souza, with approximately 1,100 inhabitants, is located 64 km from Belo Horizonte, in the metropolitan region, along one of the arms of the Brumadinho reservoir, where there is a high density of the *Anopheles darlingi* mosquito. Epidemiological investigation suggested transmission originated from a single individual (imported case) infected after visiting a malaria-endemic area in Humaitá, State of Amazonas (approximately 2,000 km away from the outbreak region). Twenty-five autochthonous cases were diagnosed, and the outbreak was promptly controlled through antimalarial treatment (chloroquine, total dose of 25 mg/kg over 3 days and primaquine, total dose of 3.5 mg/kg over 7 days) and spatial/residential application of residual insecticides (cypermethrin). No new cases since May 21, 2003, indicated the interruption of the transmission cycle.^1^ *Cuiabá municipality, Mato Grosso State (MT)*: Samples (n = 40) of patients presenting *P. vivax* malaria were collected between 2004 and 2013 at the malaria reference center of the Júlio Muller University Hospital (UFMT). The following criteria were used to select patients: a relapsed *P. vivax* malaria infection occurred between 29 days and six months; patients not re-exposed to malaria transmission during the interval between clinical malaria episodes; absence of other *Plasmodium* infections; and, if female, the absence of pregnancy. All *P. vivax*-infected patients had traveled to an endemic area of malaria, where they were infected. After returning home, they were not re-exposed to *Plasmodium* infection. Cuiabá, not being an actively malaria-transmitting area, provides an advantage for studies on relapses of *P. vivax* malaria.^5^ Individuals were treated according to guidelines of the Brazilian Ministry of Health with a combination of chloroquine (total dose, 25 mg/kg over 3 days) and primaquine (total dose, 3.5 mg/kg over 7 days). *Porto Velho municipality, Rondônia State (RO)*: Samples (n=49) were obtained from patients treated at the Tropical Medicine Research Center of Rondônia (CEPEM). Most of subjects (n=33) were enrolled in a clinical trial (2012-2015) previously published and designed in accordance with WHO guidelines.^3,6^ In brief, inclusion criteria were *P. vivax* mono-infection confirmed by microscopy, with fever or a history of fever in the previous 48 h, parasite densities > 250/μL, adults (18 to 70 years old), weight between 50 and 90 kg, and haemoglobin levels > 7.0 g/dL. Patients were randomly allocated to three treatment groups: (a) artesunate–mefloquine (100 + 200 mg QD for 3 days) (ASMQ); (b) chloroquine (CQ) (600 mg on day 1 and 450 mg on days 2 and 3); and (c) artemether–lumefantrine (20 + 120 mg BID for 3 days) (AL). All three arms received the same concomitant PRIMAQUINE regimen (7–9 days: 0.5 mg/kg/day). They were assessed on the day of enrollment and days 1, 2, 3, 7, 14, 21, 28, 42, and 63. Fourteen samples were collected in a cross-sectional study in 2019. All patients were treated following the malaria treatment guidelines of the Brazilian Ministry of Health (chloroquine, total dose of 25 mg/kg over 3 days and primaquine, total dose of 3.5 mg/kg over 7 days).

# *Malaria endemic area with high exposure*

# *Boa Vista municipality, Roraima State (RR)*: One hundred samples were collected between October 2019 and March 2020 from individuals who sought treatment at the government-operated malaria clinic, Policlínica Cosme e Silva. Approximately 70% of individuals were involved in activities associated with gold mining, a context characterized by high exposure to malaria. The enrolled participants were diagnosed with a mono-infection by *P. vivax* through microscopic examination of Giemsa-stained thick blood smears evaluated by well-trained microscopists following the malaria diagnosis guidelines of the Brazilian Ministry of Health. The prescribed treatment for the subjects followed the guidelines of the Ministry of Health, which consisted of a combination of chloroquine (total dose, 25 mg/kg over 3 days) and primaquine (total dose, 3.5 mg/kg over 7 days). These samples were collected before and 72 hours (D3) after treatment as part of a follow-up study to evaluate the effects of polymorphisms in pharmacogenes on gametocyte clearance. Primaquine and carboxyprimaquine blood levels were measured on D3.

# *Passive surveillance over 6 months*

# Malaria is an obligatory notifiable disease in Brazil. Thus, the number of *P. vivax* malaria episodes for each participant from all areas, except for the drug efficacy study (Porto Velho/RO), was obtained from the Epidemiological Surveillance System for Malaria (SIVEP-Malaria). *P. vivax* recurrence was defined as a new episode diagnosed microscopically, occurring within an interval ranging from 29 to 180 days after the initial episode. In Brazil, the first recurrence typically occurs within this interval. Overall recurrence rates were considered due to challenges in reliably distinguishing relapse from reinfection or recrudescence through clinical assessment or parasite genotyping. For participants of the drug efficacy trial, parasite relapses were assayed within 63 days of follow-up.

# Genotyping of MAO-A uVNTR Polymorphism

For the genotyping of the *MAO-A* uVNTR polymorphism, conventional PCR was performed using specific primers as described by Zhu and colleagues,^7^ followed by agarose gel visualization and capillary electrophoresis. The primer sequences used for identifying the polymorphism in the gene encoding the MAO-A enzyme were: MAOa PB1-F 5’-GAACGGACGCTCCATTACGGA-3' and MAOa PT1-R 6-FAM 5'-ACAGCCTGACCGTGGAGAAG-3’. For the reaction, we added 0.2 µL of Taq DNA Polymerase (Thermo Fisher Scientific), 500 nM of each primer, 1.5 nM of MgCl_2_, 1X Buffer, and 2 μL of DNA (10 ng/μL) in a final volume of 20 μL. The amplification conditions were: 10 min at 95ºC and 35 cycles of 1 min at 95ºC, 15 sec at 62ºC and 1 min at 72⁰C, followed by one single cycle of 5 min at 72⁰C. The reactions were carried out in the Veriti 96-well Thermal Cycler (Thermo Fisher Scientific). The results of all reactions were visualized on a 1.8% agarose gel stained with 5% v/v ethidium bromide. Subsequently, the amplified products were subjected to capillary electrophoresis on an ABI 3730 automatic sequencer (Thermo Fisher Scientific) for precise fragment size analysis using GeneScan 500 LIZ dye Size Standard (Thermo Fisher Scientific). Results were compiled and analyzed using GeneMapper v.4.1 software (Thermo Fisher Scientific).

The MAO-A activity was evaluated based on the analysis of fragment sizes obtained for each sample, following the criteria described by Sabol and colleagues.^8^ The uVNTR PCR product sizes were 286, 316, 346, and 376 bp in length, corresponding to 2R, 3R, 4R, and 5R alleles, respectively. These alleles are associated with either low (2R, 3R, and 5R with 2, 3, and 5 repeats, respectively) or high (3.5R and 4R alleles corresponding to 3.5 and 4 repeats, respectively) transcription efficiency. For the classification of heterozygous women carrying both high and low expression MAO-A alleles, it was established that they have predicted MAO-A activity as extensive metabolizers. This criterion was based on Matsusue and colleagues^9^ data, demonstrating that heterozygous women exhibited activity similar to those homozygous for the extensive allele.

***CYP2D6* Genotyping and Copy Number Analysis**

We conducted genotyping and copy number analysis to assess *CYP2D6* polymorphisms. Eight single nucleotide polymorphisms (SNPs) (C1584G, C100T, C1023T, G1846A, C2580T, G2988A, G3183A, and G4180C) and one deletion (2615_2617delAAG) within the *CYP2D6* gene, along with the gene copy number, were examined using quantitative PCR (qPCR). TaqMan SNP genotyping assays (Thermo Fisher Scientific) using specific hydrolysis probes were employed for each polymorphism. All amplification reactions followed the protocol outlined by Silvino and colleagues.^10^ Amplification and fluorescence detection were performed using the ViiA 7 real-time PCR system (Thermo Fisher Scientific). Data analysis was conducted through QuantStudio real-time PCR software (Thermo Fisher Scientific) and the Thermo Fisher cloud platform.

To determine the copy number of the *CYP2D6* gene, qPCR was employed with the Hs00010001_cn assay (Thermo Fisher Scientific) for gene deletion and amplification detection. Amplification reactions adhered to the protocol by Silvino and colleagues.^10^ Amplification and fluorescence detection took place on the ViiA 7 real-time PCR system (Thermo Fisher Scientific), with the number of copies estimated using CopyCaller v.2.0 software (Thermo Fisher Scientific). Only samples with confidence values exceeding 95% and absolute z-scores below 1.75 were considered in our analysis.

The prediction of CYP2D6 phenotype was based on the AS model.^11,12^ The inferred haplotypes were compared to the CYP2D6 haplotypes derived from the Pharmacogene Variation Consortium (PharmVar) database (<https://www.pharmvar.org/gene/CYP2D6>) for allele designation. Each allelic variant was assigned a value relative to the fully functional *CYP2D6*1* reference allele (assigned a value of 1). Nonfunctional alleles received a value of 0, while reduced activity alleles were assigned 0.5 to reflect the perceived level of activity reduction. Alleles with gene duplications or multiplications received double the value compared to a single gene copy. The sum of these values allowed classification of subjects into poor metabolizers (gPM [the prefix “g” indicates that the CYP2D6 phenotype was predicted from genotype], AS of 0), intermediate metabolizers (gIM, AS of 0.25-0.5), normal-slow metabolizers (gNM-S, AS of 1), normal-fast metabolizers (gNM-F, AS of 1.5 and AS of 2), and ultrarapid metabolizers (gUM, AS of >2). Haplotypes not matching known *CYP2D6* alleles were labeled as undetermined.

**Quantification of Primaquine and Carboxiprimaquine Levels**

Primaquine and Carboxyprimaquine were measured in a reversed-phase HPLC system with UV detection (Flexar, Perkin Elmer, Shelton, MA US™), after a previous step of separation of filter paper following by liquid-liquid extraction with methyl-ter-butyl-ether in alkaline media. The column was an RP-18 (X terra 4.6 × 150 mm, i.d. 5-μm) at 25 °C, the mobile phase was methanol and formic acid (1:3) eluted at a flow rate of 1.0 mL/min and monitored at 263nm; The limit of detection was 15 ng/mL, and the limit of quantification was 25 ng/mL for both compounds. The assay was linear from 25 to 2000 ng/mL for primaquine, and from 25 to 2500ng/mL for carboxyprimaquine. The within and between mean coefficients of variation were 12.5% and 15.5% for primaquine, and 15.1%, and 17.4% for carboxyprimaquine. The mean recovery was 82% and 81% for the parent compound and the metabolite. chloroquine, desethylchloroquine, mefloquine, carboxy-mefloquine, or acetaminophen did not interfere in the detection of the analytes.^13^

**Statistical analysis**

Proportions are given with 95% confidence intervals and compared with Fisher’s exact test. Comparison of continuous variables was performed by Kruskal–Wallis test, with Dunn’s post hoc test. All tests were two-sided, and a *P*-value less than 0.05 was considered statistically significant. Statistical analysis was performed using GraphPad Prism version 8.0.2 (GraphPad Software, San Diego, California/USA).

**REFERENCES**

1. Ceravolo IP, Sanchez BAM, Sousa TN, *et al*. Naturally acquired inhibitory antibodies to Plasmodium vivax Duffy binding protein are short-lived and allele-specific following a single malaria infection. *Clin Exp Immunol* 2009; 156:502–510.

2. Silvino ACR, Costa GL, Araújo FCF de, *et al*. Variation in Human Cytochrome P-450 Drug-Metabolism Genes: A Gateway to the Understanding of Plasmodium vivax Relapses. *PLoS One* 2016; 11:e0160172.

3. Daher A, Aljayyoussi G, Pereira D, *et al*. Pharmacokinetics/pharmacodynamics of chloroquine and artemisinin-based combination therapy with primaquine. *Malar J* 2019; **18**:325.

4. Salazar YEAR, Louzada J, Puça MCS de B, *et al*. Delayed gametocyte clearance in *Plasmodium vivax* malaria is associated with polymorphisms in the cytochrome P450 reductase (CPR). *Antimicrob Agents Chemother* 2024; **68**:e0120423.

5. Silvino ACR, Costa GL, Araújo FCF de, *et al*. Variation in Human Cytochrome P-450 Drug-Metabolism Genes: A Gateway to the Understanding of Plasmodium vivax Relapses. *PLoS One* 2016; **11**:e0160172.

6. WHO. Methods for surveillance of antimalarial drug efficacy. Geneva: World Health Organization, 2009.

7. Zhu Q, Grimsby J, Chen K, Shih J. Promoter organization and activity of human monoamine oxidase (MAO) A and B genes. *J Neurosci* 1992; **12**:4437–4446.

8. Sabol SZ, Hu S, Hamer D. A functional polymorphism in the monoamine oxidase A gene promoter. *Hum Genet* 1998; **103**:273–279.

9. Matsusue A, Kubo S, Ikeda T, *et al*. VNTR polymorphism in the monoamine oxidase A promoter region and cerebrospinal fluid catecholamine concentrations in forensic autopsy cases. *Neurosci Lett* 2019; **701**:71–76.

10. Silvino ACR, Kano FS, Costa MA, *et al*. Novel Insights into Plasmodium vivax Therapeutic Failure: CYP2D6 Activity and Time of Exposure to Malaria Modulate the Risk of Recurrence. *Antimicrob Agents Chemother* 2020; **64**:e02056-19.

11. Gaedigk A, Simon S, Pearce R, *et al*. The CYP2D6 Activity Score: Translating Genotype Information into a Qualitative Measure of Phenotype. *Clin Pharmacol Ther* 2008; **83**:234–242.

12. Gaedigk A, Sangkuhl K, Whirl-Carrillo M, *et al*. Prediction of CYP2D6 phenotype from genotype across world populations. *Genetics in Medicine* 2017; **19**:69–76.

13. Na-Bangchang K, Guirou EA, Cheomung A, Karbwang J. Determination of Primaquine in Whole Blood and Finger-Pricked Capillary Blood Dried on Filter Paper Using HPLC and LCMS/MS. *Chromatographia* 2014; **77**:561–569.
